# Human Adrenocortical Remodeling Leading to Aldosterone-Producing Cell Cluster Generation

**DOI:** 10.1155/2016/7834356

**Published:** 2016-09-18

**Authors:** Koshiro Nishimoto, Tsugio Seki, Yuichiro Hayashi, Shuji Mikami, Ghaith Al-Eyd, Ken Nakagawa, Shinya Morita, Takeo Kosaka, Mototsugu Oya, Fumiko Mitani, Makoto Suematsu, Yasuaki Kabe, Kuniaki Mukai

**Affiliations:** ^1^Department of Uro-Oncology, Saitama Medical University International Medical Center, Hidaka, Japan; ^2^Department of Biochemistry, Keio University School of Medicine, Shinjuku-ku, Japan; ^3^Department of Medical Education, School of Medicine, California University of Science and Medicine, Colton, CA, USA; ^4^Division of Diagnostic Pathology, Keio University School of Medicine, Shinjuku-ku, Japan; ^5^Department of Urology, Ichikawa General Hospital, Tokyo Dental College, Ichikawa, Japan; ^6^Department of Urology, Keio University School of Medicine, Shinjuku-ku, Japan; ^7^Medical Education Center, Keio University School of Medicine, Shinjuku-ku, Japan

## Abstract

*Background*. The immunohistochemical detection of aldosterone synthase (CYP11B2) and steroid 11*β*-hydroxylase (CYP11B1) has enabled the identification of aldosterone-producing cell clusters (APCCs) in the subcapsular portion of the human adult adrenal cortex. We hypothesized that adrenals have layered zonation in early postnatal stages and are remodeled to possess APCCs over time.* Purposes*. To investigate changes in human adrenocortical zonation with age. *Methods*. We retrospectively analyzed adrenal tissues prepared from 33 autopsied patients aged between 0 and 50 years. They were immunostained for CYP11B2 and CYP11B1. The percentage of APCC areas over the whole adrenal area (AA/WAA, %) and the number of APCCs (NOA, APCCs/mm^2^) were calculated by four examiners. Average values were used in statistical analyses. *Results*. Adrenals under 11 years old had layered zona glomerulosa (ZG) and zona fasciculata (ZF) without apparent APCCs. Some adrenals had an unstained (CYP11B2/CYP11B1-negative) layer between ZG and ZF, resembling the rat undifferentiated cell zone. Average AA/WAA and NOA correlated with age, suggesting that APCC development is associated with aging. Possible APCC-to-APA transitional lesions were incidentally identified in two adult adrenals.* Conclusions*. The adrenal cortex with layered zonation remodels to possess APCCs over time. APCC generation may be associated with hypertension in adults.

## 1. Introduction

Immunohistochemical detection of aldosterone synthase (CYP11B2) and steroid 11*β*-hydroxylase (CYP11B1: cortisol synthesizing enzyme) has enabled the identification of aldosterone-producing cell clusters (APCCs) in the subcapsular portion of the normal human adrenal cortex. APCCs are indistinguishable from the normal adrenal cortex on hematoxylin-eosin- (H&E-) stained adrenal sections because APCCs consist of subcapsular zona glomerulosa- (ZG-) like cells and inner zona fasciculata- (ZF-) like cells. APCCs are distinct from aldosterone-producing adenomas (APAs), which cause primary aldosteronism (PA), in the following aspects [1, 2]: (i) APCCs are approximately 0.2–1.5 mm in length, whereas APAs are more than ~3 mm in length. (ii) APCCs appear histologically normal in H&E staining with ZG- and ZF-like cells, whereas APAs consist of heterogeneous cells. (iii) APCCs express CYP11B2, but not CYP11B1, whereas APAs consist of heterogeneous tumor cells expressing either CYP11B2 or CYP11B1. Thus, APCCs and APAs are distinctive in their sizes, cellular arrangements, and enzyme expression profiles.

Previous studies demonstrated that APAs frequently harbor a somatic mutation in one of the 4 ion channel/pump genes (APA-associated mutations) including the potassium channel, inwardly rectifying subfamily J, member 5 (*KCNJ5*) [3]. These mutations are considered to cause autonomous aldosterone production by triggering cellular depolarization and/or increasing intracellular calcium concentrations. Nishimoto et al. recently reported that nearly half of the APCCs harbor APA-associated mutations [4], suggesting that APCCs produce aldosterone autonomously and are precursors of APAs. This is supported by cases of PA caused by possible APCC-to-APA transitional lesions (pAATLs), which consist of a subcapsular APCC-like portion and inner APA-like portion [2]. Nanba et al. also reported that multiple APCCs may cause PA [5]. Thus, there is accumulating evidence to show that APCCs play important roles in the pathophysiology of PA.

To date, most APCC studies have been performed using adult samples, and there has not yet been a systematical study on the relationship between age and the onset of APCCs. Therefore, in the present study, we hypothesized that human adrenal glands have conventional layered functional zonation similar to that of rat adrenal glands [6, 7] in the early postnatal stages and are remodeled to adrenals with APCCs over time. We herein investigated changes in human adrenocortical zonation using archival adrenal samples from autopsied cases.

## 2. Materials and Methods

### 2.1. Ethics

This study was approved by the Medical Ethics Committee of the Keio University School of Medicine (approval #20090018).

### 2.2. Sample Collection

Based on autopsy records, we retrospectively selected 52 specimens of adrenal tissue prepared from 48 autopsied patients aged between 0 and 50 years (Supplementary Table 1, in Supplementary Material available online at http://dx.doi.org/10.1155/2016/7834356). However, 17 samples from 15 cases (32.7%) were excluded from analyses because of their weak or absent immunostaining (*∗* in Supplementary Table 1). Poor staining was probably attributed to a decrease in antigenicity due to issues after sample excision (e.g., prolonged storage). Consequently, 35 samples (20 male and 15 female samples) from 33 cases (18 male and 15 female cases) were subjected to analyses (Table 1). Two pathologists (YH and SM) confirmed that these adrenals had no pathological nodules on H&E-stained specimens.

### 2.3. Immunohistochemistry for CYP11B2 and CYP11B1

Adrenal sections from formalin-fixed paraffin-embedded samples were double-immunostained for CYP11B2 with 5-bromo-4-chloro-3-indolyl-phosphate/nitro blue tetrazolium (blue color) and CYP11B1 with 3,3′-diaminobenzidine (brown color) as previously reported [2].

### 2.4. Estimation of APCC Areas and Number of APCCs over the Whole Adrenal Area

Images of CYP11B2/CYP11B1-stained adrenal sections were printed out at 800% the original size (Supplementary Figure 1). Four examiners from the authors (two physicians [KN and TS], a biochemist [KM], and a pathologist [GAE]) independently identified APCCs on stained glass slides under a microscope and marked them on the printouts. The “width” (length of APCCs along the adrenal capsule) and “depth” (distance from the capsule end to the farthest point in a vertical direction) of each APCC were calculated from measurements on the printouts. All APCCs were numbered, and the area of each APCC was calculated, assuming that all APCCs have a semiellipse shape, by *π* (3.14) × “width” × “depth”/4. As for the whole adrenal area, scanned adrenal images were traced using Photoshop software version 13.0 (Adobe Systems, San Jose, CA), and their sizes were measured with ImageJ 1.50e software. Of note, areas of the medulla and blood vessels were not subtracted from the whole adrenal area. The percentage of the sum of APCC areas over the whole adrenal area (AA/WAA, %) and the number of APCCs (NOA, APCCs/mm^2^) were calculated and used in statistical analyses.

### 2.5. Statistical Analysis

Comparisons of AA/WAA and NOA among 4 examiners were analyzed by the Kruskal-Wallis one-way analysis of variance on ranks. The relationship between AA/WAA and NOA between examiners and that between age and AA/WAA or NOA were analyzed by Spearman's rank order correlation test. *p* values less than 0.05 were considered significant.

## 3. Results

**Figure 1:**
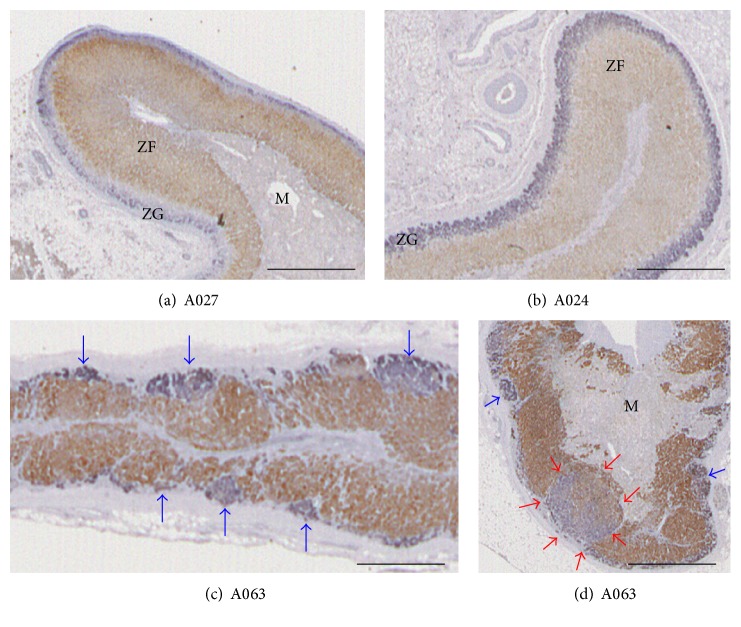
Double immunohistochemistry for CYP11B2 (blue) and CYP11B1 (brown). (a) Section A027 from Case 7; (b) section A024 from Case 4; (c) section A063 from Case 40; (d) section A063 from Case 40. These are enlarged images of frames in Supplementary Figure 1. Bars indicate 1 mm. Blue and red arrows indicate APCCs and pAATL, respectively.

Double-immunostaining for CYP11B2 and CYP11B1 was performed successfully on 35 samples (adrenal sample#: A021-072) from 33 cases (Cases 1–48, Table 1, Supplementary Figure 1). All adrenals from 0- to 11-year-old individuals (Cases 1–8) showed clearly layered zonation both histologically and immunohistochemically (Table 1, Figures 1(a) and 1(b), Pages 1-2 of Supplementary Figure 1). Some of these samples had an unstained layer between CYP11B2-positive ZG and CYP11B1-positive ZF, which appeared to be immunohistochemically similar to the undifferentiated cell zone (ZU) found in rat adrenal glands (Figure 1(a)) [6, 8]. The A024 sample from a 1.8-year-old male infant (Case 4) had a very thick CYP11B2-positive ZG layer, which was similar to the adrenals of a rat fed with a sodium-deficient diet (Figure 1(b)) [6, 8]. Case 4 underwent liver transplantation and may have developed hyponatremia prior to his death (clinical data was not available). Overall, the adrenals from children aged 11 or younger had conventional adrenocortical layers and lacked APCCs.

On the other hand, we frequently observed APCCs in the samples obtained from adults aged between 18 and 50 years (Table 1, Figure 1(c), Pages 3–9 of Supplementary Figure 1). In order to examine APCC areas, four investigators (KN, KM, TS, and GAE) independently measured AA and NOA. Variations among examiners were primarily due to disagreements related to small APCC, which some examiners judged to be APCC and others as irregular ZG. Hence, the AA/WAA (%) and NOA (APCCs/mm^2^) values from the 4 examiners were slightly different (*p* = 0.756 and 0.696, resp.; Kruskal-Wallis one-way analysis of variance on ranks). AA/WAA values from the different examiners were correlated with each other (*r* values for KN versus KM, KN versus TS, KN versus GAE, KM versus TS, KM versus GAE, and TS versus GAE were 0.96, 0.90, 0.92, 0.95, 0.94, and 0.95, resp.; *p* < 0.001 each). Similarly, NOA from the different examiners were correlated with each other (*r* values for KN versus KM, KN versus TS, KN versus GAE, KM versus TS, KM versus GAE, and TS versus GAE were 0.95, 0.89, 0.85, 0.96, 0.91, and 0.91, resp.; *p* < 0.001 each). These results indicated that the AA and NOA measurements by the different examiners were essentially in agreement.

**Figure 2:**
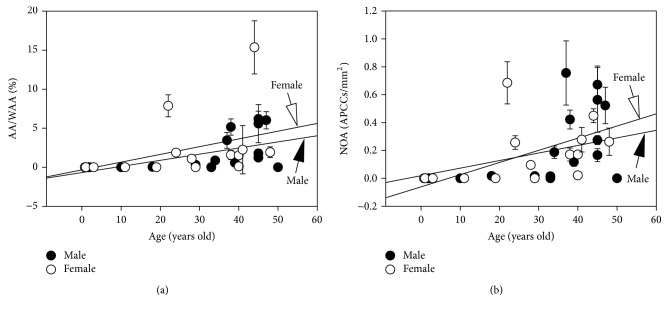
Relationship between age versus AA/WAA (%, (a)) and age versus NOA (APCCs/mm^2^, (b)). Error bars indicate SEM between 4 examiners (see Table 1 and Supplementary Table 1). Regression lines of females and males are drawn. AA/WAA: percentage of APCC areas over the whole adrenal area (%). NOA: number of APCCs/mm^2^.

The AA/WAA value (median [25%–75% interquartile range]: 1.52% [0.07–4.30]) and NOA value (0.17 [0.02–0.44]) from individuals aged between 18 and 50 years were significantly higher than those from children aged between 0 and 11 years (0.00% [0.00–0.00] and 0.00 [0.00–0.00]/mm^2^, respectively; *p* < 0.001 each; Mann-Whitney rank sum test). The average values of AA/WAA and NOA in each sample correlated with age (*r* = 0.65, *p* < 0.001 and *r* = 0.64, *p* < 0.001, resp.; Spearman's rank order correlation). The AA/WAA-age correlation was observed in the male and female groups (Figure 2(a); *r* = 0.67, *p* < 0.01 for male group and *r* = 0.71, *p* < 0.01 for female group). Similarly, the NOA-age correlation was observed in male and female groups (Figure 2(b); *r* = 0.61, *p* < 0.01 for male group and *r* = 0.69, *p* < 0.01 for female group). These results indicated that APCCs developed in adulthood and generally increased in size and number with aging in males and females.

We detected pAATLs in 2 adult cases, which consisted of a subcapsular APCC-like-portion and inner APA-like portion (red arrows in Figure 1(d) and a red arrowhead in page 3 [A036] and page 8 [A063] of supplemental Figure  1. In the analyzed cases, detailed clinical records were discarded, and the autopsy records did not show if individuals with pAATLs had PA.

## 4. Discussion

In the present study, we demonstrated for the first time that APCCs develop postnatally, mostly during adulthood, and their sizes and numbers increase with aging. Furthermore, adrenals of infant to child appear to have a negative layer for CYP11B2 and CYP11B1.

As previously reported, APCCs frequently harbor APA-associated mutations [4]. Cell lineage analyses using genetically engineered mice have shown that some progenitor cells in the adrenal capsule generate ZG cells [9], which develop further into ZF cells [10]. One of our hypotheses on APCC generation is that a mutation in ZG cells blocks ZG to ZF transdifferentiation, which causes the accumulation of mutant ZG cells and, thus, APCC formation [4]. Alternatively, APCCs may develop due to aging, contemporary diet, or other environmental factors, and the excessive production of aldosterone from APCCs may induce APA-associated mutations in APCCs.

We previously reported a novel undifferentiated cell zone (ZU) in the rat adrenal cortex between ZG and ZF, which is devoid of Cyp11b2 and Cyp11b1 [8]. The ZU was not clearly recognizable in mice, and a difference was observed among rat strains; Wistar rats have markedly thinner ZU than Sprague-Dawley rats [11]. The double-negative zone in rats is physically fragile, and the capsular portion (adrenal capsule and ZG) may be separated from the inner adrenal zones (ZF, ZR, and medulla) manually along the double-negative zone. Enucleation surgery leaving the capsular portion results in the regeneration of ZF and ZR beneath the ZU [12], suggesting that ZF cells differentiate from the ZU or the ZU comprises transitional/intermediate cells between ZG and ZF cells. The results of the present study showed that the human adult adrenals seldom had ZU, which may be due to aging-related adrenocortical remodeling.

## 5. Conclusions

Collectively, we have described adrenal functional zonation throughout postnatal development, that is, infant, child, and adult adrenals. We and our collaborators have identified novel PA pathologies including APCCs and pAATL [1, 2, 5]. The findings of these studies suggest that APCCs are a key initial pathological event for autonomous aldosterone production and hyperaldosteronism. Therefore, further studies on APCCs are needed in order to elucidate the pathophysiology of PA and develop novel treatments, including less invasive surgery such as APCC ablation and/or curative pharmacological treatments that target APCCs.

## Supplementary Material

Supplementary Figure 1. Immunohistochemistry for CYP11B2 (blue) and CYP11B1 (brown) in all analyzed cases. Each image is labeled with the case number and section number. Red arrowheads indicate pAATLs.
Supplementary Table 1: Text in bold font has already been presented in Table 1, and additional information is shown in standard font. Additional information includes AA/WAA and NOA/WAA values measured by each examiners as well as patient characteristics of excluded samples.

## Figures and Tables

**Table 1:** Patient characteristics, AA/WAA, and NOA/WAA.

Case #	Section #	Age	Sex	Cause of death	AA/WAA (%)	NOA/WAA (APCCs/mm^2^)
1	A021	0.75	F	Postliver transplantation	0.000 ± 0.000	0.000 ± 0.000
2	A022	1	M	Heterotaxy syndrome	0.000 ± 0.000	0.000 ± 0.000
3	A023	1	F	Brain tumor	0.000 ± 0.000	0.000 ± 0.000
4	A024	1.8	M	Postliver transplantation	0.000 ± 0.000	0.000 ± 0.000
5	A025	2	M	Acute lung dysfunction	0.000 ± 0.000	0.000 ± 0.000
6	A026	3	F	Pulmonary dysfunction	0.000 ± 0.000	0.000 ± 0.000
7	A027	10	M	Encephalopathy due to Reye's syndrome	0.000 ± 0.000	0.000 ± 0.000
8	A028	11	F	Malignant peripheral nerve sheath tumors	0.000 ± 0.000	0.000 ± 0.000
10	A030	18	M	Hemophagocytic syndrome	0.042 ± 0.042	0.017 ± 0.017
12	A032	19	F	Postliver transplantation	0.000 ± 0.000	0.000 ± 0.000
14	A034	22	F	Malignant intrapelvic tumor	7.875 ± 1.410	0.685 ± 0.151
16	A036	24	F	Pulmonary hypertension	1.871 ± 0.242	0.257 ± 0.049
19	A039	28	F	Pulmonary alveolar hemorrhage	1.094 ± 0.284	0.096 ± 0.018
21	A042	29	M	Sepsis due to acute myelocytic leukemia	0.315 ± 0.192	0.016 ± 0.016
22	A043	29	F	Subarachnoid hemorrhage	0.000 ± 0.000	0.000 ± 0.000
23	A044, A045	33	M	Sepsis	0.000 ± 0.000	0.000 ± 0.000
24	A046	33	M	Acute heart failure	0.016 ± 0.016	0.015 ± 0.015
25	A047	34	M	Aorta stenosis	0.865 ± 0.159	0.188 ± 0.044
27	A049	38	F	Sepsis	1.568 ± 0.579	0.171 ± 0.047
28	A050	38	M	Liver cirrhosis	5.156 ± 1.045	0.422 ± 0.068
29	A051	39	M	Myelodysplastic syndrome	0.576 ± 0.046	0.117 ± 0.030
30	A052	40	F	Gastric cancer	1.520 ± 0.399	0.171 ± 0.019
31	A053	40	F	Pulmonitis due to systemic lupus erythematosus	0.104 ± 0.104	0.021 ± 0.021
32	A054, A055	41	F	Ovarian carcinoma	2.250 ± 3.099	0.277 ± 0.088
36	A059	44	F	Renal cell carcinoma	15.362 ± 3.400	0.449 ± 0.050
37	A060	45	M	Heart failure	1.229 ± 0.194	0.274 ± 0.032
38	A061	45	M	Lung cancer	1.771 ± 0.530	0.166 ± 0.047
40	A063	45	M	Hepatic failure due to liver cancer	6.197 ± 0.996	0.672 ± 0.134
42	A065	45	M	Heart failure	5.587 ± 2.451	0.563 ± 0.232
43	A066	47	M	Bronchiectasis	6.022 ± 1.113	0.523 ± 0.131
44	A067	48	F	Pulmonary hypertension	1.921 ± 0.689	0.263 ± 0.098
47	A071	50	M	Heart failure	0.000 ± 0.000	0.000 ± 0.000
48	A072	37	M	Acute pancreatitis	3.449 ± 1.018	0.756 ± 0.230

F: female; M: male; AA/WAA: percentage of APCC areas over the whole adrenal area; NOA: number of APCCs/mm^2^. Each value of AA/WAA and NOA by individual examiners and the characteristics of cases not used in statistical analyses are shown in Supplementary Figure 1.
