# Supplementary material for: Human Adrenocortical Remodeling Leading to Aldosterone-Producing Cell Cluster Generation
Source: Int J Endocrinol. 2016 Sep 18;2016:7834356. doi: 10.1155/2016/7834356 (PMC5046023; doi:10.1155/2016/7834356)
Supplement: Supplementary file 1 — Supplementary Figure 1. Immunohistochemistry for CYP11B2 (blue) and CYP11B1 (brown) in all analyzed cases. Each image is labeled with the case number and section number. Red arrowheads indicate pAATLs. Supplementary Table 1: Text in bold font has already been presented in Table 1, and additional information is shown in standard font. Additional information includes AA/WAA and NOA/WAA values measured by each examiners as well as patient characteristics of excluded samples. [file 7834356.f1.pdf]

Supplementary Figure 1

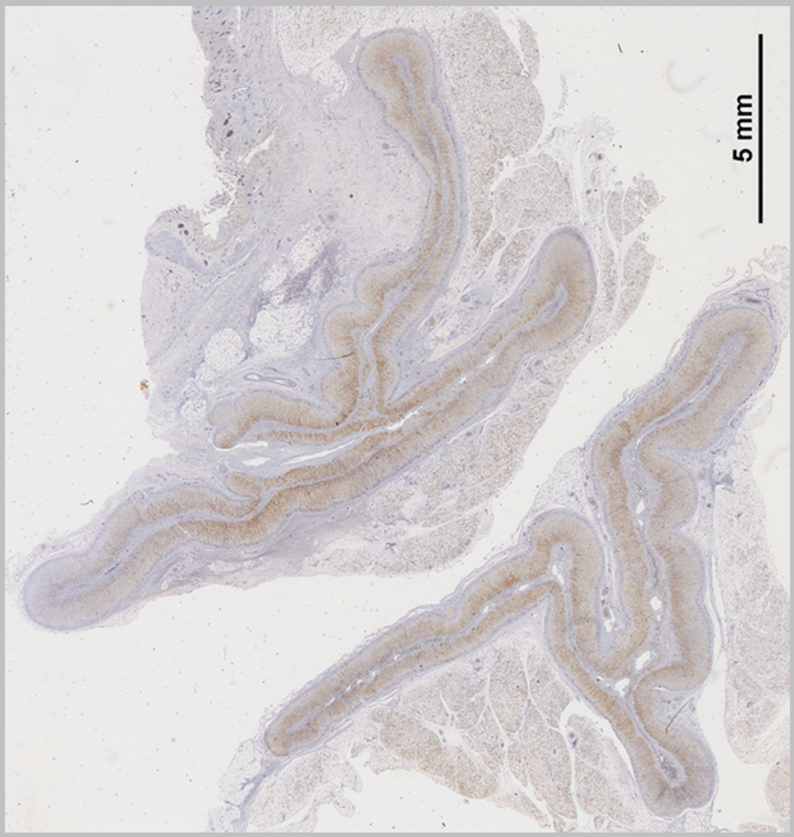

Case 1, A021

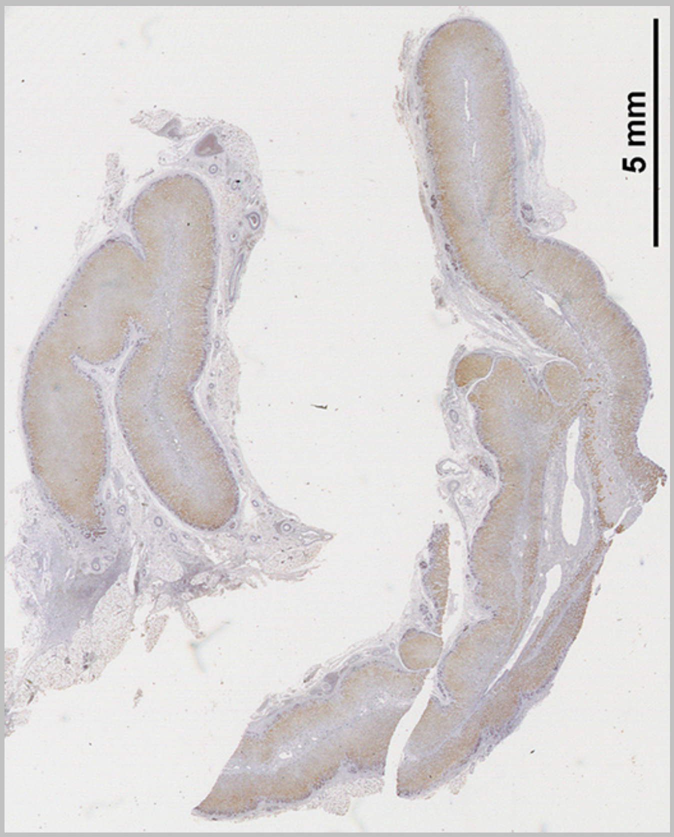

Case 2, A022

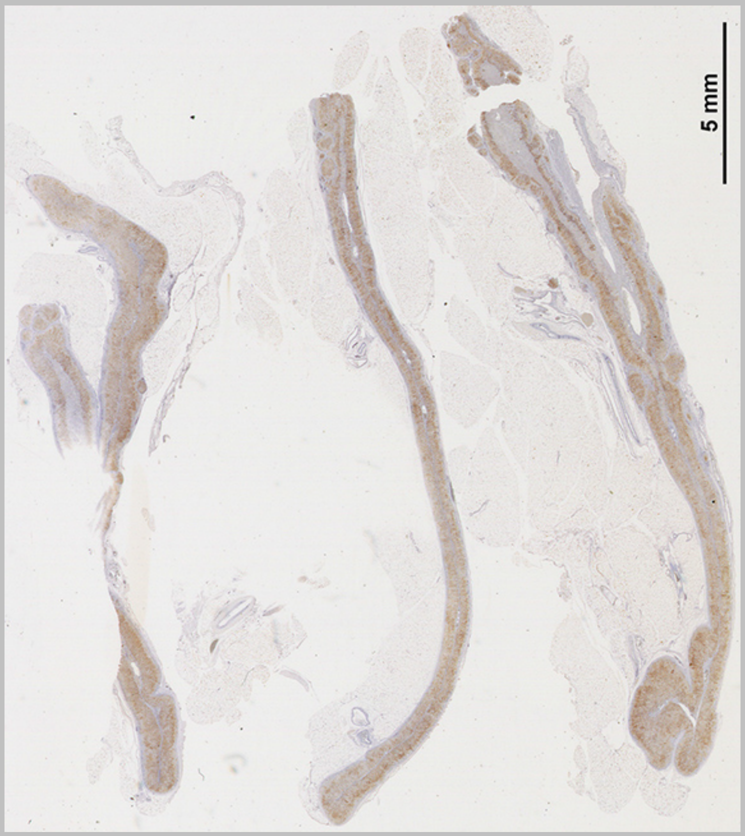

Case 3, A023

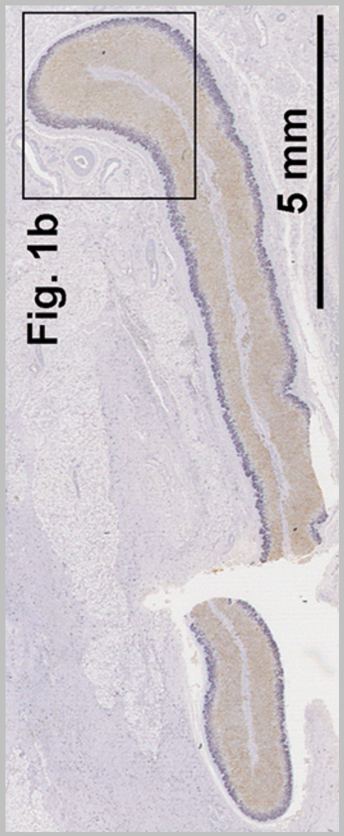

Case 4, A024

Supplementary Figure 1

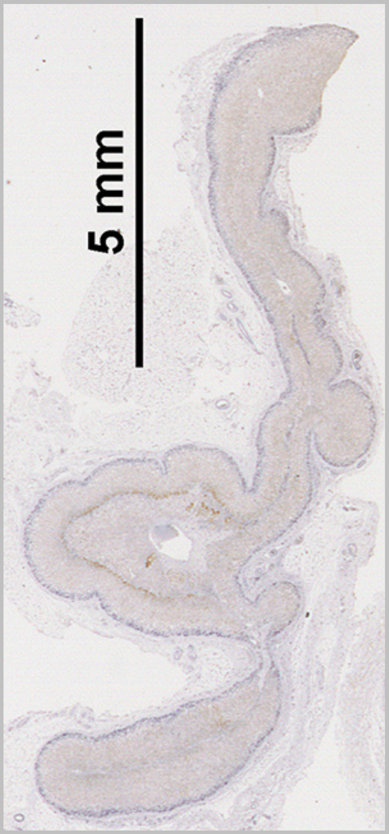

Case 5, A025

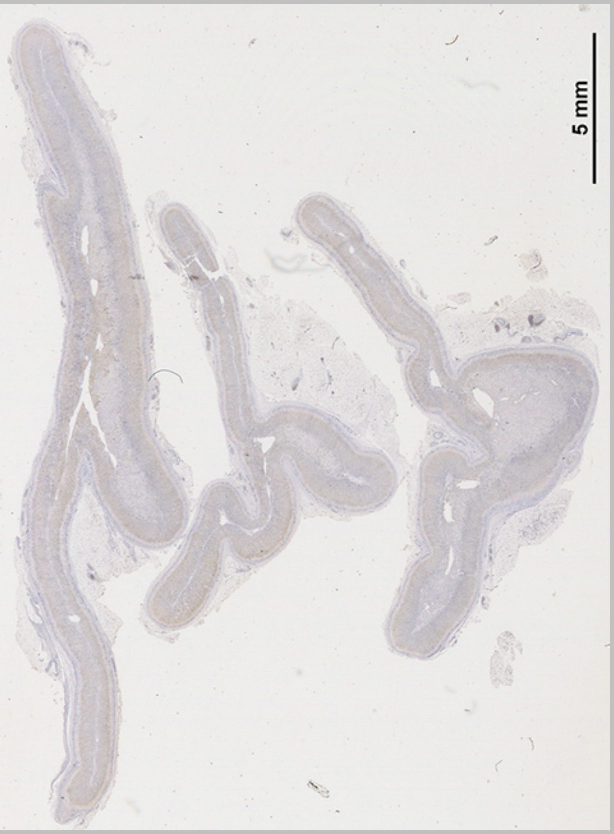

Case 6, A026

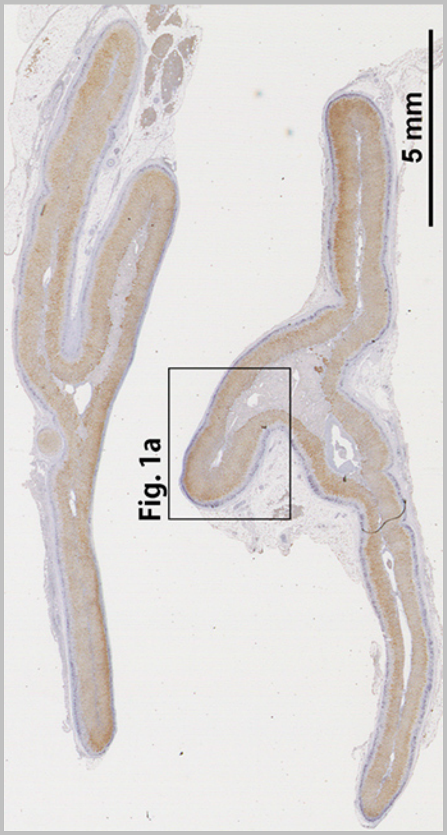

Case 7, A027

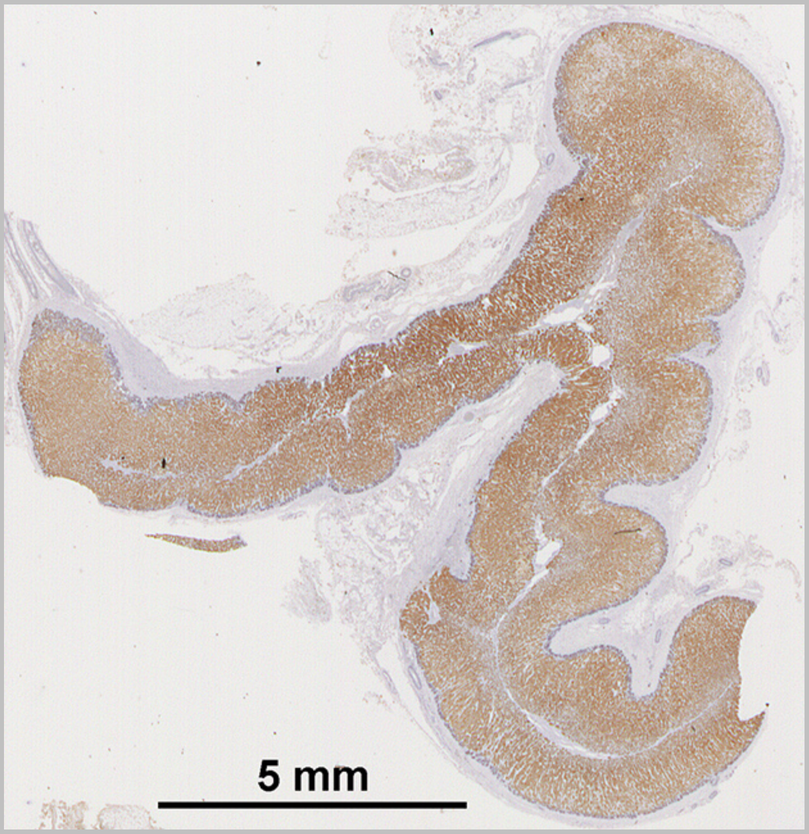

Case 8, A028

Supplementary Figure 1

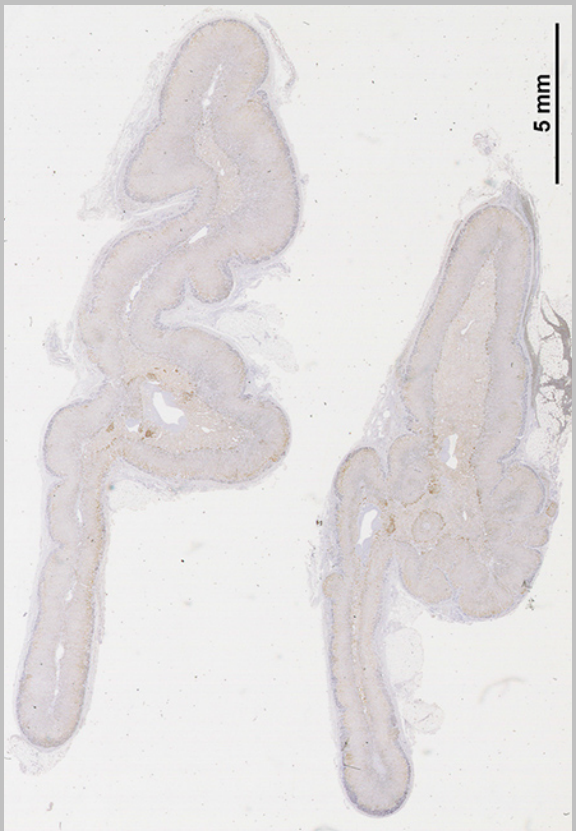

Case 10, A030

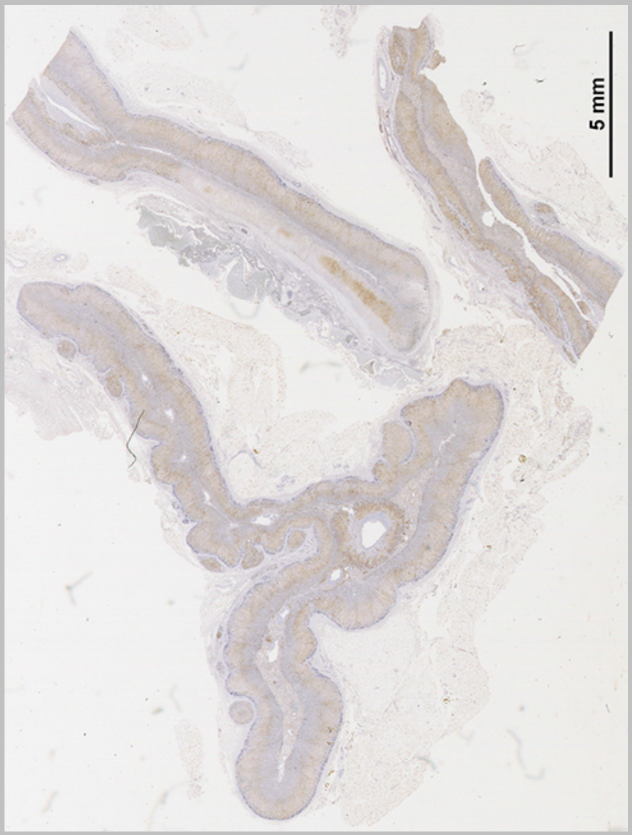

Case 12, A032

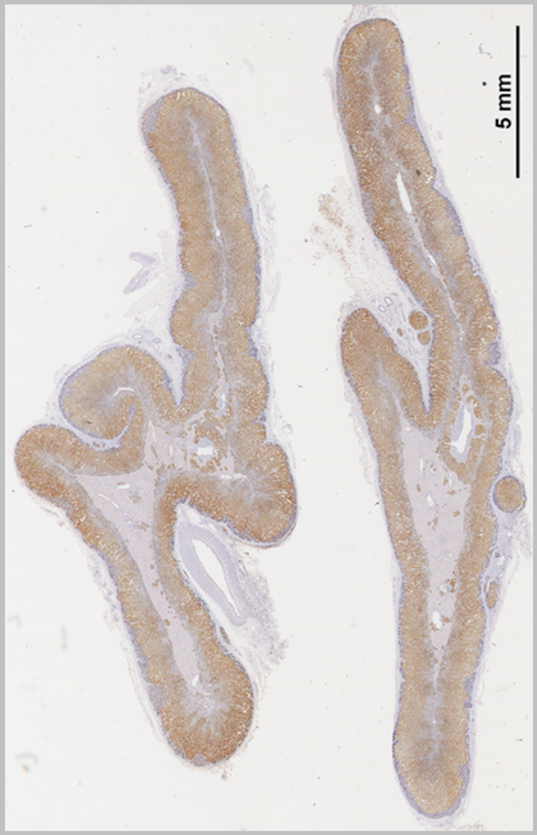

Case 14, A034

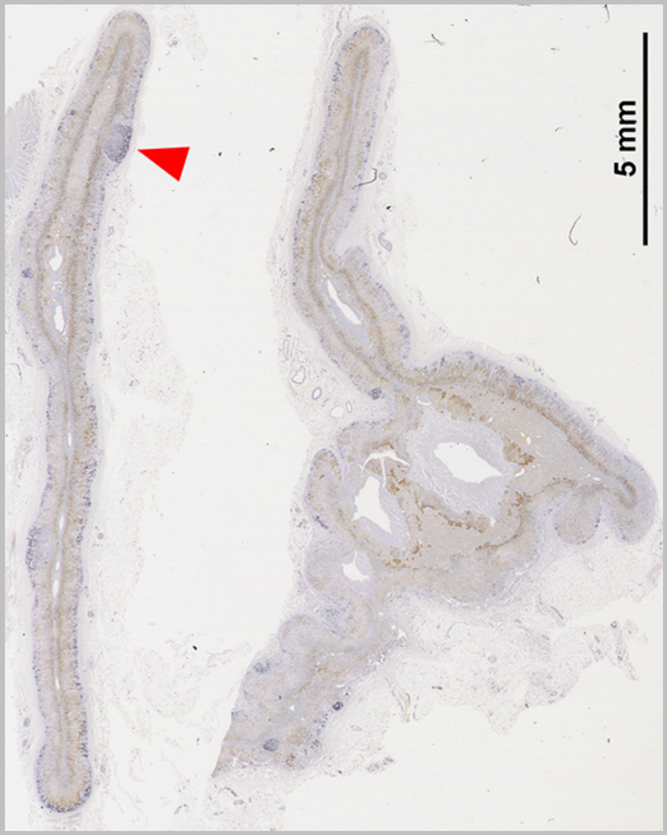

Case 16, A036

Supplementary Figure 1

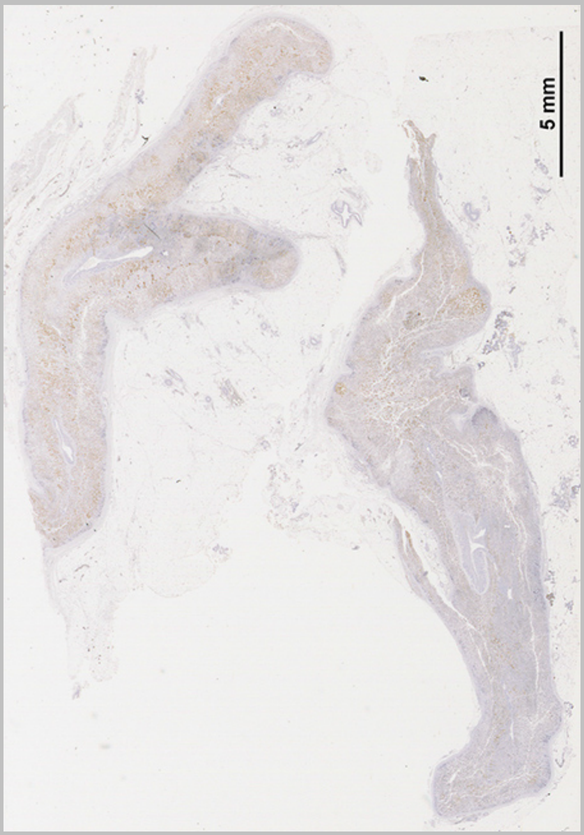

Case 19, A039

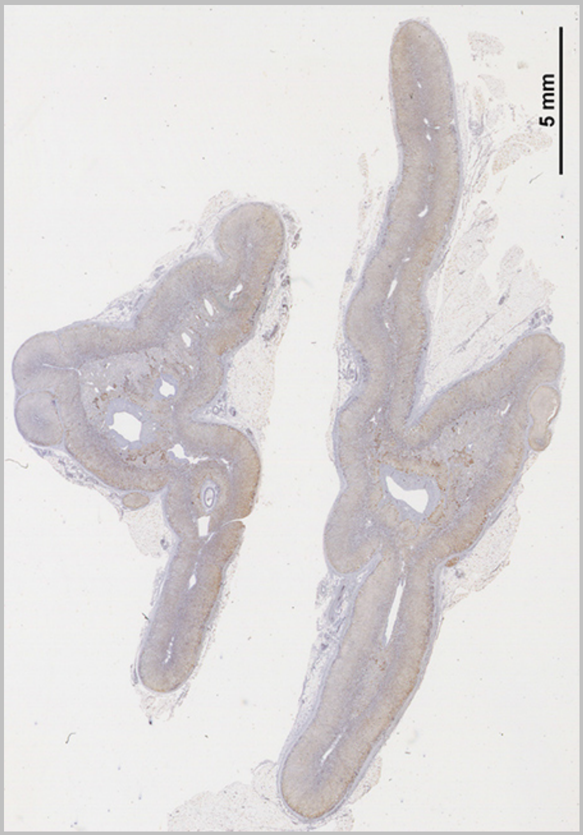

Case 21, A042

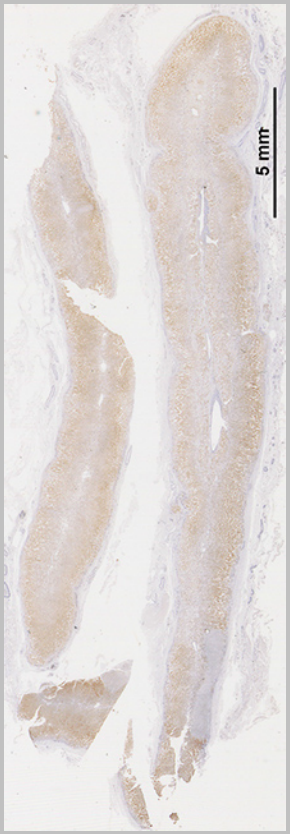

Case 22, A043

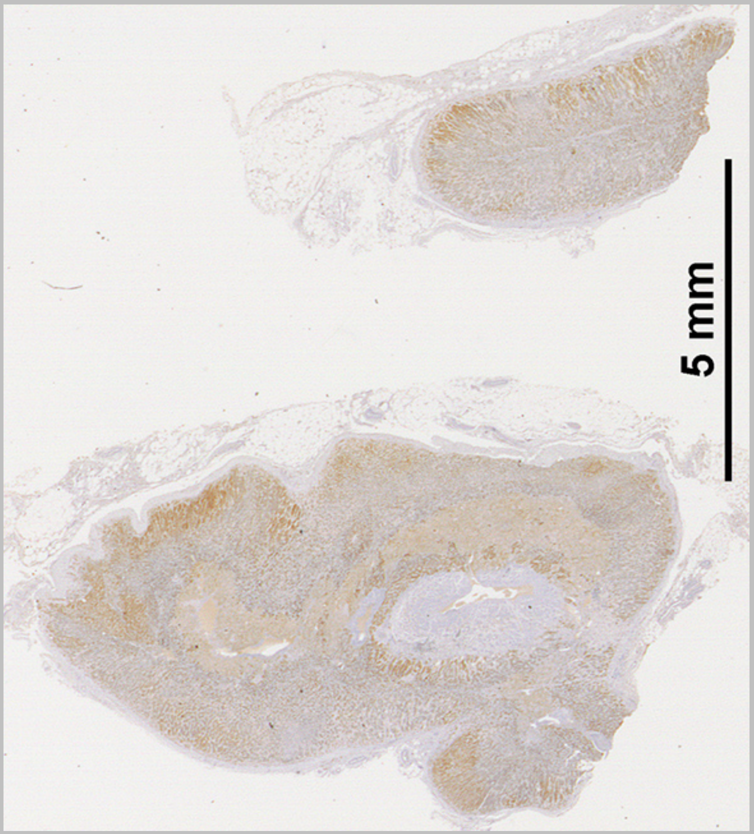

Case 23, A044

Supplementary Figure 1

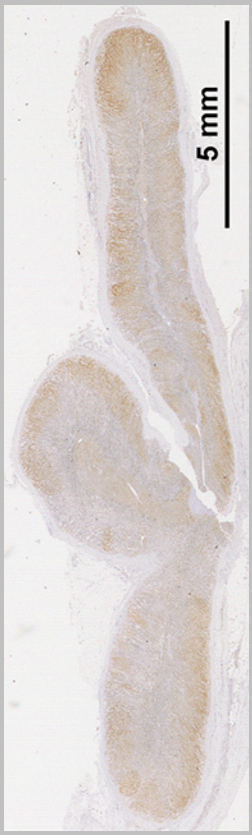

Case 23, A045

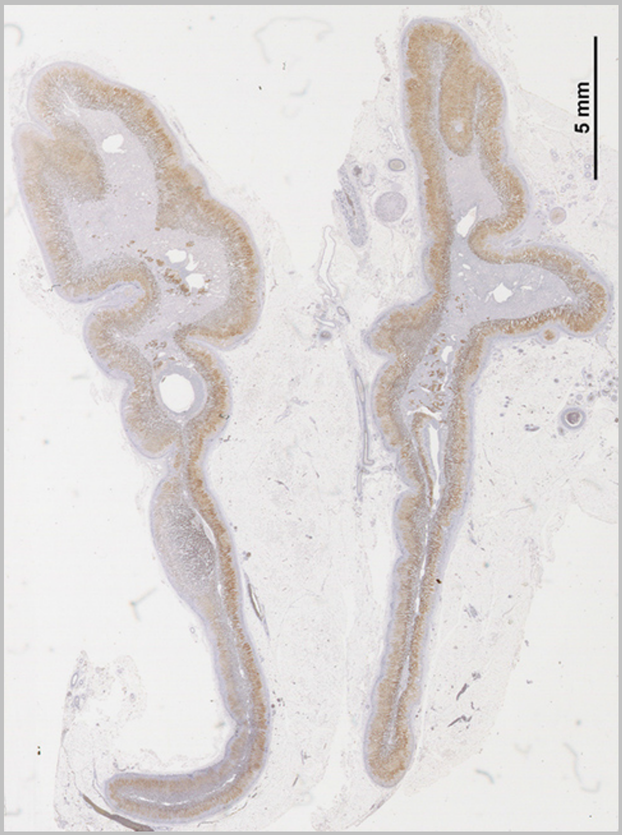

Case 24, A046

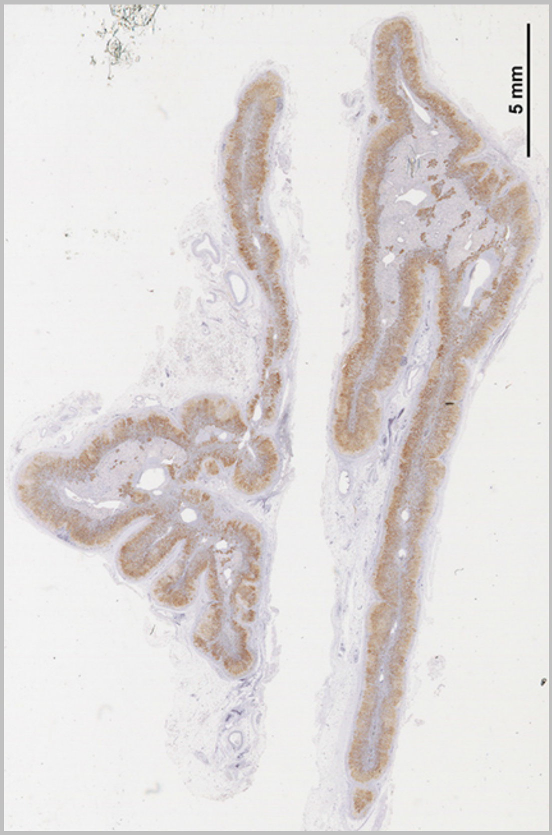

Case 25, A047

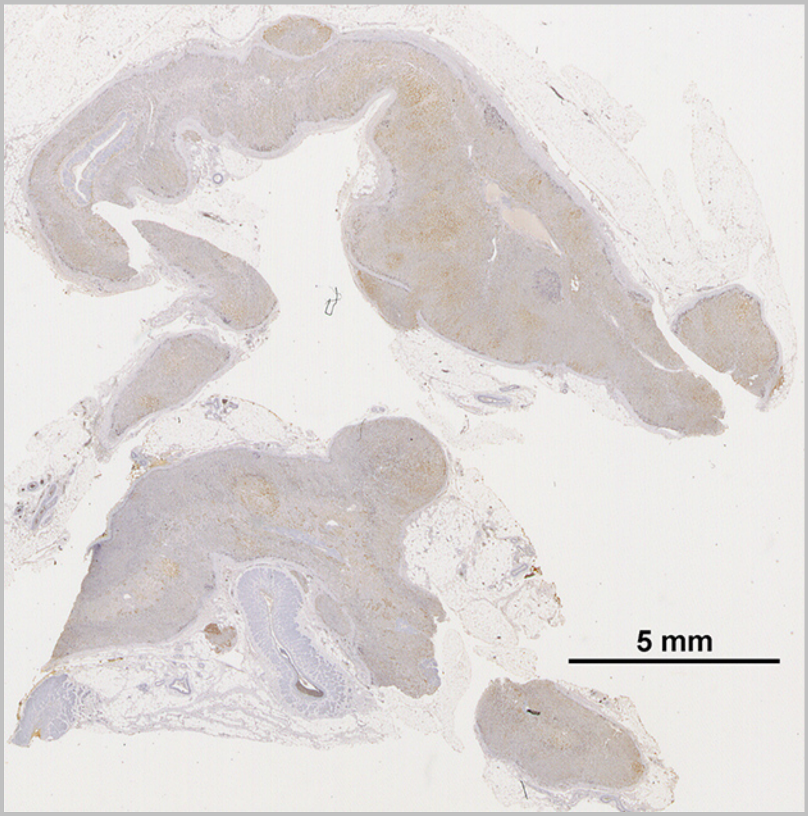

Case 27, A049

Supplementary Figure 1

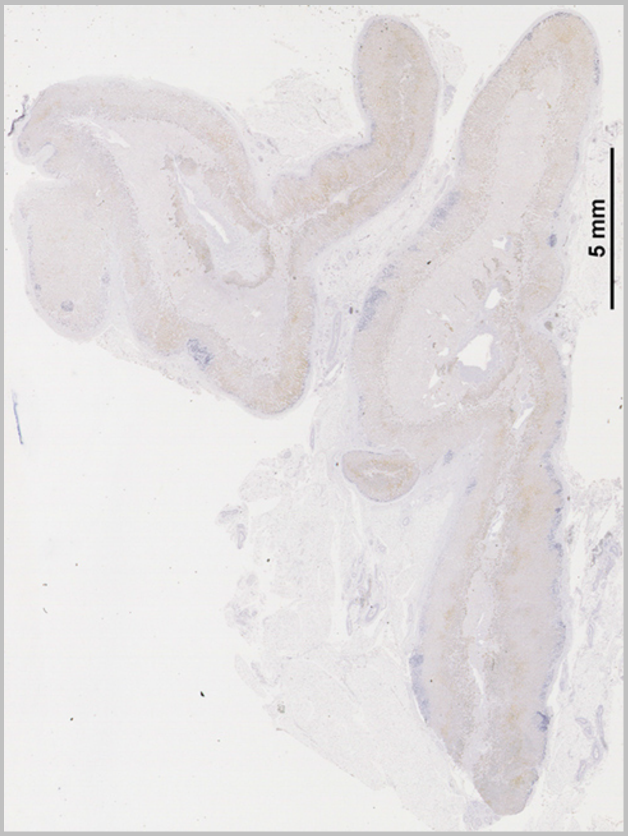

Case 28, A050

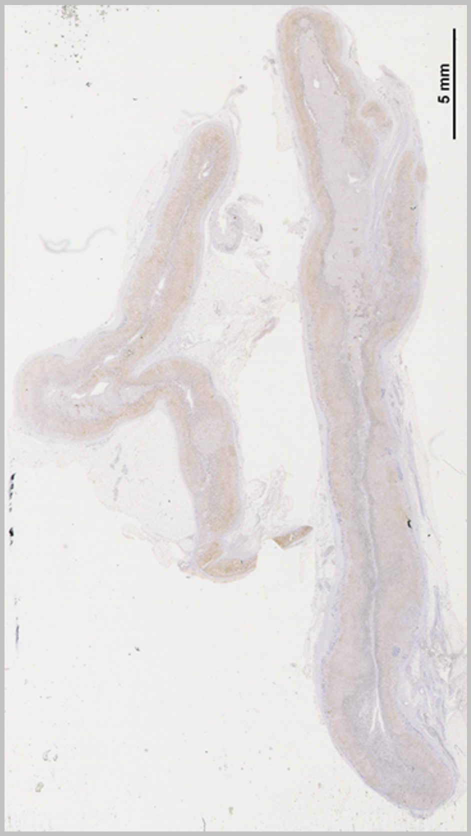

Case 29, A051

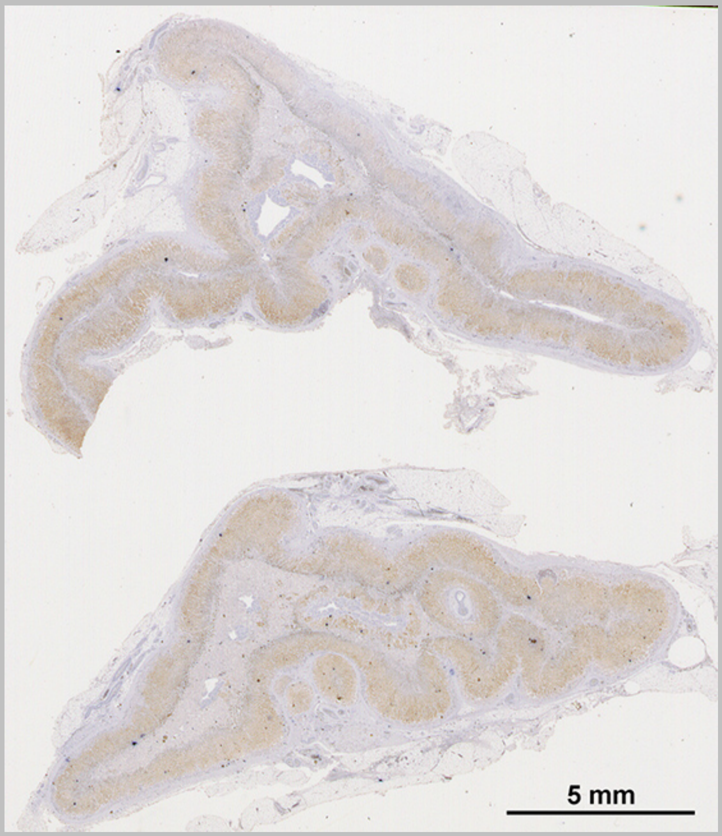

Case 30, A052

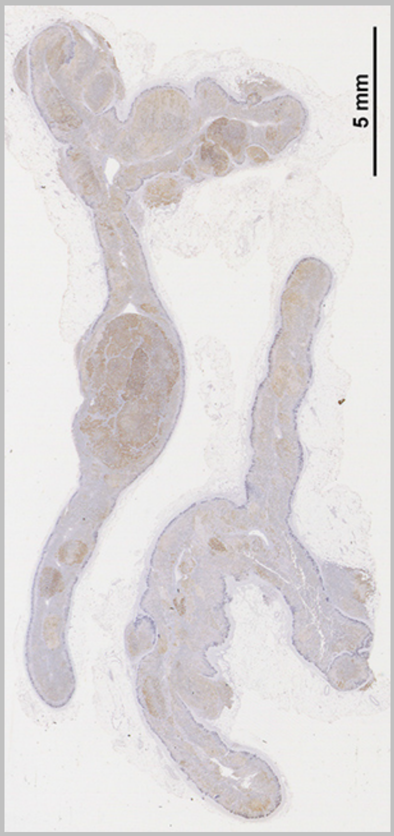

Case 31, A053

Supplementary Figure 1

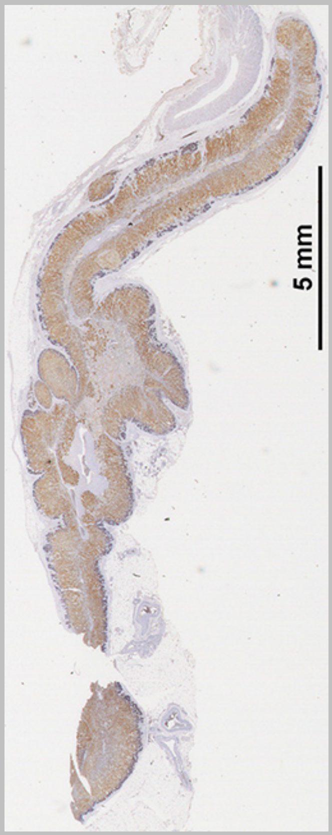

Case 32, A054

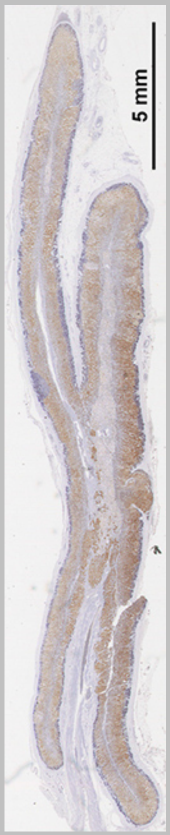

Case 32, A055

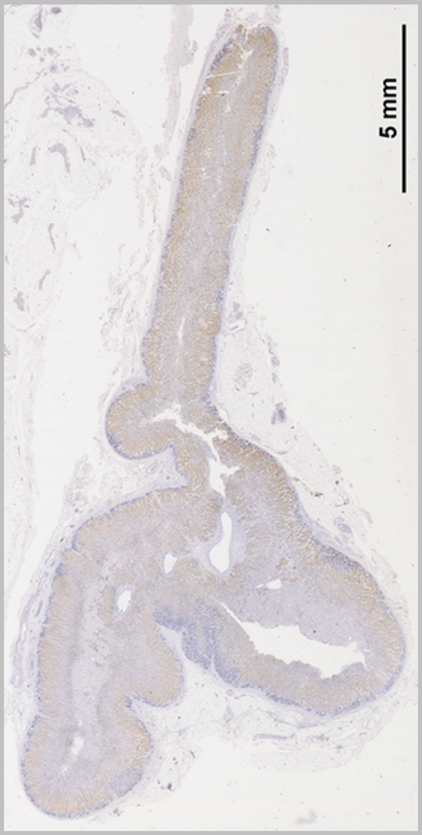

Case 36, A059

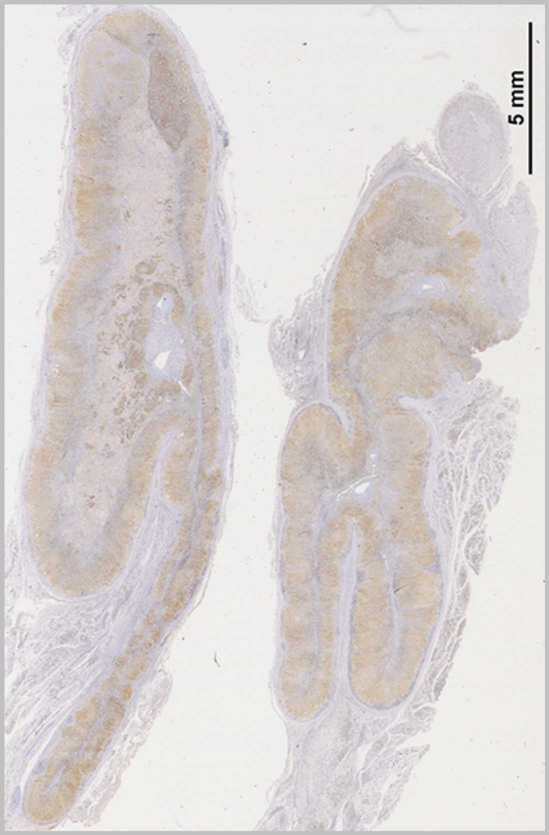

Case 37, A060

Supplementary Figure 1

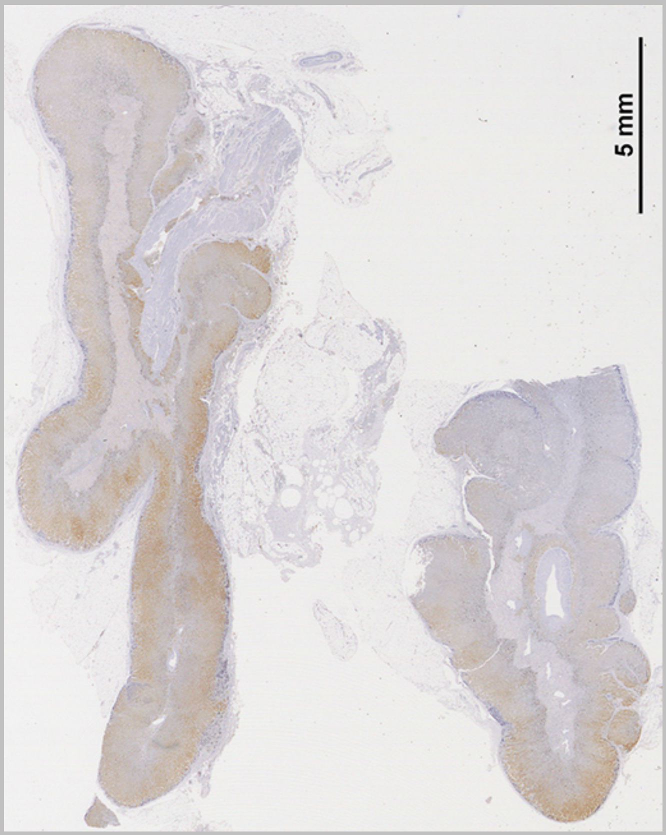

Case 38, A061

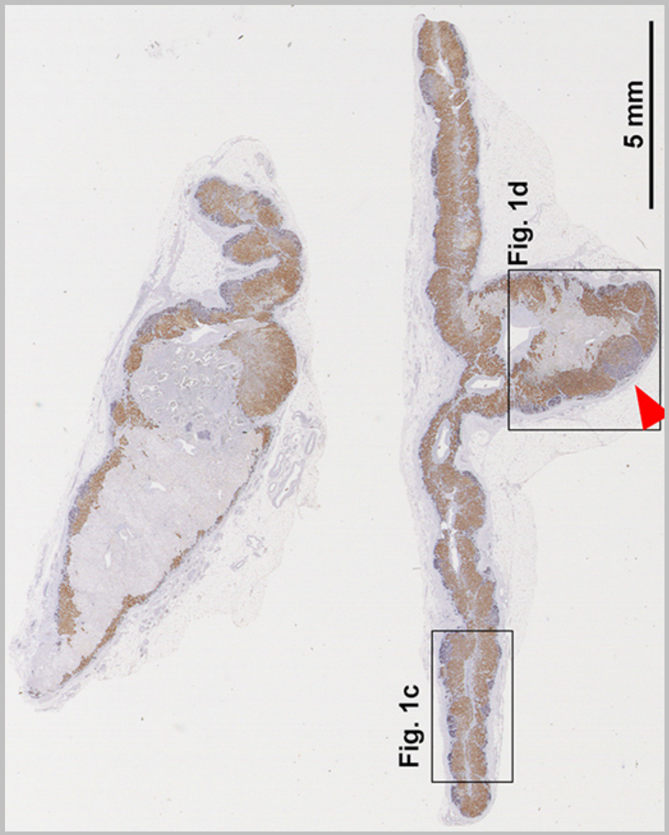

Case 40, A063

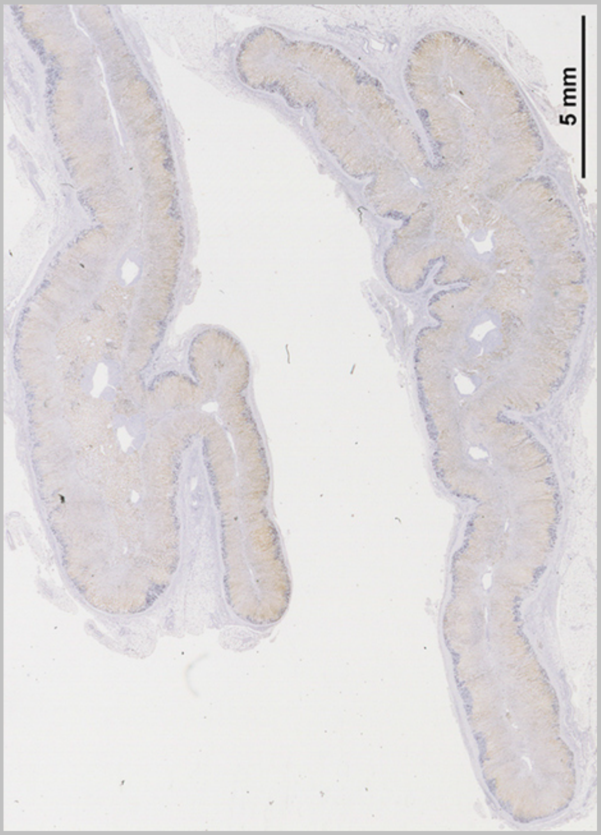

Case 42, A065

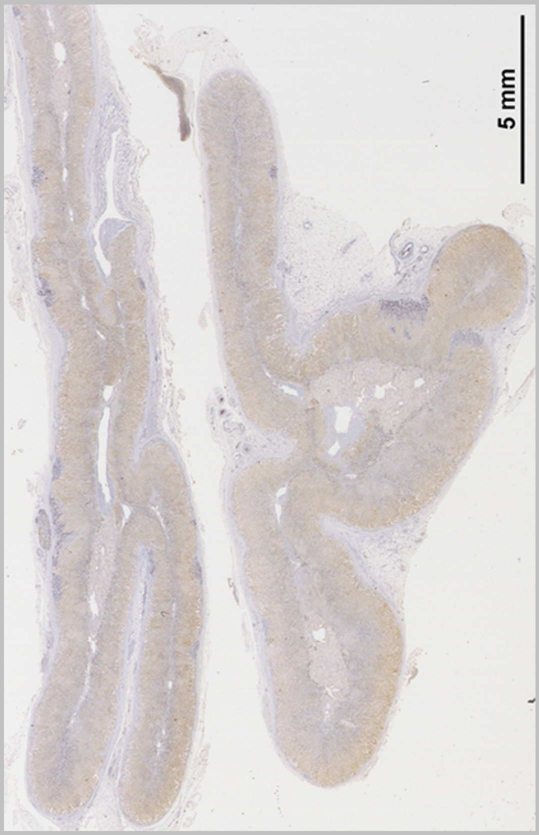

Case 43, A066

Supplementary Figure 1

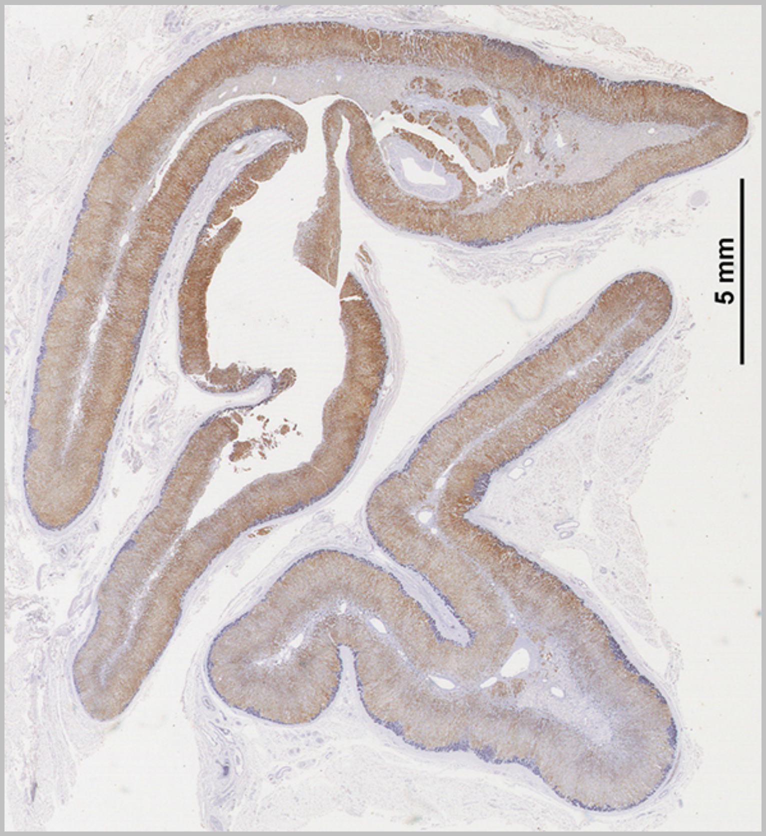

Case 44, A067

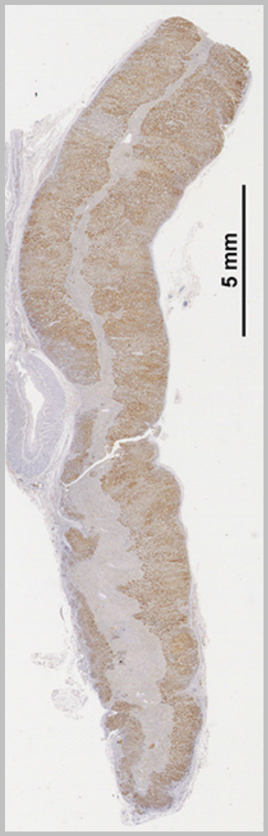

Case 47, A071

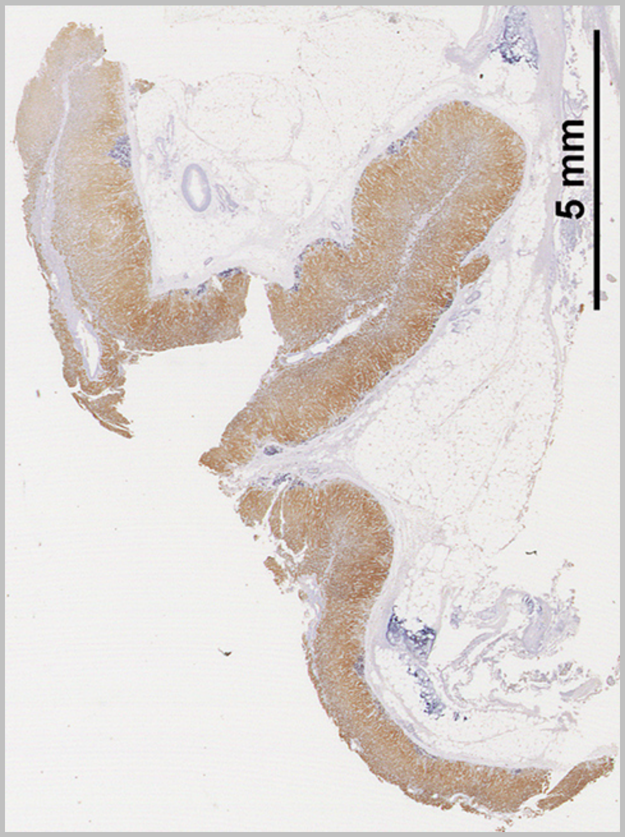

Case 48, A072

Supplementary Table 1.

Case#	Section#	Age	Sex	Cause of death	KN AA/WAA (%)	KN NOA/ WAA (mm^2)	KM AA/WAA (%)	KM NOA/ WAA (mm^2)	TS AA/WAA (%)	TS NOA/ WAA (mm^2)	GEA AA/WAA (%)	GEA NOA/ WAA (mm^2)	Average AA/WAA (%)	Average NOA/ WAA (mm^2)
1	A021	0.75	F	post liver transplantation	0.000	0.000	0.000	0.000	0.000	0.000	0.000	0.000	0.000 ± 0.000	0.000 ± 0.000
2	A022	1	M	heterotaxy syndrome	0.000	0.000	0.000	0.000	0.000	0.000	0.000	0.000	0.000 ± 0.000	0.000 ± 0.000
3	A023	1	F	brain tumor	0.000	0.000	0.000	0.000	0.000	0.000	0.000	0.000	0.000 ± 0.000	0.000 ± 0.000
4	A024	1.8	M	post liver transplantation	0.000	0.000	0.000	0.000	0.000	0.000	0.000	0.000	0.000 ± 0.000	0.000 ± 0.000
5	A025	2	M	acute lung dysfunction	0.000	0.000	0.000	0.000	0.000	0.000	0.000	0.000	0.000 ± 0.000	0.000 ± 0.000
6	A026	3	F	pulmonary dysfunction	0.000	0.000	0.000	0.000	0.000	0.000	0.000	0.000	0.000 ± 0.000	0.000 ± 0.000
7	A027	10	M	encephalopathy due to Reye's syndrome	0.000	0.000	0.000	0.000	0.000	0.000	0.000	0.000	0.000 ± 0.000	0.000 ± 0.000
8	A028	11	F	malignant peripheral nerve sheath tumors	0.000	0.000	0.000	0.000	0.000	0.000	0.000	0.000	0.000 ± 0.000	0.000 ± 0.000
* 9	A029	17	F	liver cirrhosis										
10	A030	18	M	hemophagocytic syndrome	0.000	0.000	0.000	0.000	0.169	0.069	0.000	0.000	0.042 ± 0.042	0.017 ± 0.017
* 11	A031	19	M	pulmonary hypertension										
12	A032	19	F	post liver transplantation	0.000	0.000	0.000	0.000	0.000	0.000	0.000	0.000	0.000 ± 0.000	0.000 ± 0.000
* 13	A033	22	M	myocarditis										
14	A034	22	F	malignant intrapelvic tumor	5.324	0.298	9.941	0.894	10.663	0.954	5.573	0.596	7.875 ± 1.410	0.685 ± 0.151
* 15	A035	23	M	acute renal failure										
16	A036	24	F	pulmonary hypertension	1.922	0.233	1.653	0.280	2.520	0.373	1.392	0.140	1.871 ± 0.242	0.257 ± 0.049
* 17	A037	24	M	brain hemorrhage										
* 18	A038	24	M	acute myelocytic leukemia										
19	A039	28	F	pulmonary alveolar hemorrhage	1.412	0.128	0.294	0.064	1.569	0.128	1.099	0.064	1.094 ± 0.284	0.096 ± 0.018
* 20	A040, A0	28	M	sepsis										
21	A042	29	M	sepsis due to acute myelocytic leukemia	0.000	0.000	0.000	0.000	0.485	0.000	0.777	0.066	0.315 ± 0.192	0.016 ± 0.016
22	A043	29	F	subarachnoid hemorrhage	0.000	0.000	0.000	0.000	0.000	0.000	0.000	0.000	0.000 ± 0.000	0.000 ± 0.000
23	A044, A0	33	M	sepsis	0.000	0.000	0.000	0.000	0.000	0.000	0.000	0.000	0.000 ± 0.000	0.000 ± 0.000
24	A046	33	M	acute heart failure	0.000	0.000	0.000	0.000	0.066	0.060	0.000	0.000	0.016 ± 0.016	0.015 ± 0.015
25	A047	34	M	aorta stenosis	0.795	0.250	0.723	0.188	1.327	0.250	0.614	0.063	0.865 ± 0.159	0.188 ± 0.044
* 26	A048	36	F	brain tumor										
27	A049	38	F	sepsis	3.237	0.292	0.860	0.097	0.718	0.097	1.459	0.195	1.568 ± 0.579	0.171 ± 0.047
28	A050	38	M	liver cirrhosis	3.550	0.369	6.115	0.474	7.646	0.580	3.312	0.264	5.156 ± 1.045	0.422 ± 0.068
29	A051	39	M	myelodysplastic syndrome	0.637	0.140	0.660	0.187	0.458	0.093	0.550	0.047	0.576 ± 0.046	0.117 ± 0.030
30	A052	40	F	gastric cancer	0.895	0.152	0.770	0.152	2.162	0.228	2.255	0.152	1.520 ± 0.399	0.171 ± 0.019
31	A053	40	F	pulmonitis due to systemic lupus erythemat	0.000	0.000	0.000	0.000	0.000	0.000	0.415	0.085	0.104 ± 0.104	0.021 ± 0.021
32	A054, A0	41	F	ovarian carcinoma	1.575	0.085	2.318	0.256	3.696	0.512	1.411	0.256	2.250 ± 3.099	0.277 ± 0.088
* 33	A056	42	F	heart failure										
* 34	A057	43	M	convulsion, respiration failure										
* 35	A058	44	F	uterine sarcoma										
36	A059	44	F	renal cell carcinoma	20.350	0.399	10.896	0.399	21.947	0.599	8.255	0.399	15.362 ± 3.400	0.449 ± 0.050
37	A060	45	M	heart failure	1.683	0.329	0.899	0.219	1.420	0.329	0.915	0.219	1.229 ± 0.194	0.274 ± 0.032
38	A061	45	M	lung cancer	0.967	0.074	0.747	0.148	2.661	0.296	2.709	0.148	1.771 ± 0.530	0.166 ± 0.047
* 39	A062	45	M	acute leukemia										
40	A063	45	M	hepatic failure due to liver cancer	6.128	0.563	8.023	1.000	7.194	0.750	3.444	0.375	6.197 ± 0.996	0.672 ± 0.134
* 41	A064	45	M	C type hepatitis										
42	A065	45	M	heart failure	0.804	0.051	3.470	0.358	12.272	1.126	5.803	0.716	5.587 ± 2.451	0.563 ± 0.232
43	A066	47	M	bronchiectasis	5.379	0.339	6.231	0.621	8.916	0.847	3.564	0.282	6.022 ± 1.113	0.523 ± 0.131
44	A067	48	F	pulmonary hypertension	0.804	0.137	1.629	0.137	3.923	0.548	1.328	0.228	1.921 ± 0.689	0.263 ± 0.098

*	45	A068	49	M	myelodysplastic syndrome														
*	46	A069, A0[illegible]	50	M	gastric carcinoma														
	**47**	**A071**	**50**	**M**	**heart failure**	0.000	0.000	0.000	0.000	0.000	0.000	0.000	0.000	**0.000**	±	**0.000**	**0.000**	±	**0.000**
	**48**	**A072**	**37**	**M**	**acute pancreatitis**	2.290	0.504	3.283	1.008	6.368	1.259	1.855	0.252	**3.449**	±	**1.018**	**0.756**	±	**0.230**

*: removed samples due to low quality staining. Bold characters are shown in Table 1. Each value of AA/WAA and NOA/WAA analyzed by KN, KM, TS, and GEA is shown.
